# Utility of Surgeon-Performed Ultrasound Assessment of the Lateral Neck for Metastatic Papillary Thyroid Cancer

**DOI:** 10.1155/2012/973124

**Published:** 2012-01-15

**Authors:** Cortney Y. Lee, Samuel K. Snyder, Terry C. Lairmore, Sean C. Dupont, Daniel C. Jupiter

**Affiliations:** Section of Endocrine Surgery, Department of Surgery, Texas A&M Health Science Center and Scott & White Healthcare, 2401 S. 31st Street, Temple, TX 76508, USA

## Abstract

Ultrasound is the recommended staging modality for papillary thyroid cancer. Surgeons proficient in US assessment of the neck and experienced in the management of papillary thyroid cancer (PTC) appear uniquely qualified to assess the lateral cervical lymph nodes for metastatic disease. Of 310 patients treated for PTC between 2000 and 2008, 109 underwent surgeon-performed ultrasound (SUS) of the lateral neck preoperatively. Fine needle aspiration was performed on suspicious lateral lymph nodes. SUS findings were compared with FNA cytology and results of postoperative imaging studies. The sensitivity and negative predictive value of SUS were 88% and 97%, respectively. Four patients were found to have missed metastatic disease within 6 months. No patient underwent a nontherapeutic neck dissection. SUS combined with US-guided FNA of suspicious lymph nodes can accurately stage PTC to reliably direct surgical management.

## 1. Introduction

Papillary thyroid cancer (PTC) is the most common endocrine malignancy, and its incidence is increasing [1–3]. An exceptional cure rate and low recurrence rate begin with appropriate surgical treatment. The extent of surgery is determined by lymph node involvement of the central and lateral neck. Lateral neck disease discovered preoperatively leads to more aggressive surgery, which includes compartment-oriented lymph node dissection. Complete surgical resection of all imageable neck disease at the initial operation decreases likelihood of future surgery for recurrent disease and may impact survival.

According to current guidelines, all patients undergoing thyroidectomy for malignancy should undergo preoperative neck ultrasound (US) of the thyroid and central and lateral neck lymph basins, followed by fine needle aspiration (FNA) of suspicious lymph nodes [4]. This practice has been widely accepted and supported by leading centers [5–11].

As technology improves and physicians of a variety of specialties become more comfortable with ultrasound, its use is becoming more common across specialties. Surgeons routinely perform ultrasound in multiple arenas including trauma, breast, and vascular surgery [12–16]. In the endocrine surgery literature, much has been published about the accuracy of surgeon-performed ultrasound of thyroid nodules and parathyroid adenomas [17–22]. Although a great deal of data is available on accuracy of radiology-performed ultrasound of the lateral neck for papillary thyroid cancer staging, little is published on the accuracy of surgeon-performed ultrasound of the lateral neck.

Surgeons who treat thyroid cancer routinely and already perform neck ultrasound as part of the physical exam seem uniquely qualified to evaluate the lateral neck in patients with papillary cancer. We hypothesize that surgeon-performed ultrasound is a reliable method of evaluating the lateral neck to stage papillary thyroid cancer.

## 2. Methods

An IRB-approved retrospective chart review was performed of all patients treated for papillary thyroid cancer at Scott & White Clinic in Temple, TX, from 2000 to 2008. Patients who underwent surgeon-performed ultrasound of the lateral neck at the time of diagnosis were included in the study. The preoperative SUS evaluation was systematically done either at the time of the initial diagnosis of papillary thyroid cancer, while the patient was still on the FNA procedure table or at the initial outpatient endocrine surgeon consultation for a recently diagnosed papillary thyroid cancer. Demographic data including age, gender, body mass index (BMI), and weight were recorded. All ultrasounds were performed by one of two endocrine surgeons in the clinic setting. 96% of the ultrasound examinations were done by the first endocrine surgeon (2000–2008) and 4% by the second endocrine surgeon (2004–2008). The portable ultrasound equipment used varied over the study period including an Acusan with a 12-megahertz small-part probe (2000–2004), a Toshiba Nemio 2 with a 14-megahertz small-part probe (2005–2008), and a Sonosite with a 13-megahertz small-part probe (2007–2008). All of these ultrasound machines allowed accurate real-time imaging of lateral cervical lymph nodes.

Surgical clinic notes were reviewed to determine results of lateral neck ultrasound. Each side of the neck was treated as an individual event. Nodes were considered suspicious based on size (any dominant node, typically greater than 1 cm), shape (rounded), loss of hyperechoic stripe (fatty hilum), and presence of calcifications or cystic changes. Color Doppler assessment of cervical lymph nodes was not added as part of the examination until late in the study. FNA of a suspicious lymph node was done using 3 or 4 passes with 25 gauge needles and ultrasound guidance to sample the most suspicious areas of the lymph node and also different areas of the lymph node. These samples were immediately processed by a pathology technician and then promptly interpreted by a staff cytopathologist in the same procedure room for adequacy of the FNA sample, as well as to provide a diagnosis. When FNA was performed based on ultrasound suspicion, the lateral neck was defined as ultrasound-positive. Ultrasound findings were compared to cytopathologic results of FNA. If both were positive, it was recorded as a true positive. If ultrasound was positive and FNA negative, it was recorded as a false positive.

Postoperative imaging was used to evaluate the ultrasound-negative lateral neck. Short-term imaging consisted of radioactive iodine scans immediately before or after radioactive iodine ablation. Long-term followup consisted primarily of periodic radiology-performed ultrasound of the lateral neck followed by FNA of any suspicious nodes. Persistent (missed) disease was classified as disease discovered in an ultrasound-negative neck within 6 months of surgery. True negatives were ultrasound-negative necks that had no evidence of persistent disease. False negatives were ultrasound-negative necks in which disease was discovered within 6 months of surgery.

## 3. Results

Of 310 patients with papillary thyroid cancer treated during the time period, 109 met inclusion criteria. The majority of exclusions were due to lack of surgeon-performed ultrasound of the lateral neck for two primary reasons: incidental diagnosis of PTC on final pathology and initial surgery and evaluation performed at an outside hospital. PTC was incidentally diagnosed in thyroid specimens obtained after thyroidectomy for other indications, and almost all tumors were less than 1 cm in size. Some patients in the early years of the study did not undergo surgeon-performed ultrasound as it was a newer diagnostic option for the surgeon. Seven patients were lost to follow up before any postsurgical imagining was obtained. Average length of followup was 34.1 (±23.9) months.

Patient characteristics revealed 75.5% female, average age 54.1 years (±16.4), and average BMI 29.4 (±5.3). Average primary tumor size was 1.85 cm (±1.2), and 43 (41%) of patients had multifocal PTC. Average size of lymph nodes evaluated by 111 FNAs was 1.37 cm (±0.53). Twenty-eight modified radical neck dissections were performed on 27 patients after the initial SUS assessment. Metastatic papillary thyroid cancer was confirmed on final pathology in all 28 dissections (Table 1).

Postoperative RAI scans were performed on almost all patients (92%) at an average of 2.28 months after surgery. Four patients were found to have persistent (missed) disease in the lateral neck—three discovered with postoperative RAI scans and one with ultrasound. All but one were confirmed with pathologic evaluation. (One of the four patients had clearly evident lateral neck disease on posttreatment RAI scan but failed to return to clinic for pathologic confirmation or additional surgical treatment.) Three patients had recurrent disease discovered at 9, 19, and 30 months. These patients were classified as recurrences, not persistence, and were therefore evaluated as true negatives. Ultrasound was the primary imaging study used for long-term surveillance.

Sensitivity, specificity, and positive and negative predictive values of SUS of the lateral neck were 88%, 68%, 33%, and 97%, respectively, (Table 2). All false-positive ultrasound results were proven negative by US-guided FNA. No patient underwent a neck dissection based on a false-positive ultrasound result. All patients with positive FNA results indicating metastatic papillary thyroid cancer underwent therapeutic lateral modified radical neck lymph node dissection. Metastatic papillary cancer was confirmed in all of the surgical specimens.

## 4. Discussion

Ultrasound is the recommended imaging modality for preoperative staging of PTC [4]. Appropriate staging is important not only for predicting outcome but for directing surgical resection. Complete resection of all imageable disease in the neck during the initial surgery is the best way to prevent reoperative surgery due to persistent or recurrent disease.

Much has been published on the accuracy of ultrasound for staging of the lateral neck in papillary thyroid cancer [5–11]. Ultrasound imaging is traditionally performed by radiology, not the operating surgeon. Surgeons who routinely treat PTC and perform neck ultrasound and FNA are uniquely qualified to stage the lateral neck at the time of diagnosis. The results of this study support surgeon-performed ultrasound of the lateral neck in patients with papillary thyroid cancer.

We report both higher sensitivity (88%) and negative predictive value (97%) than other published studies of radiology-performed ultrasound. Published sensitivities of ultrasound of the lateral neck in PTC range from 51 to 83.5% in radiology-performed studies [6, 23, 24] and as high as 93.8% in one surgeon-performed ultrasound study [11]. Higher sensitivity was due to liberal FNA of any dominant or suspicious lymph node. All false-positive ultrasound findings were proven negative by FNA. As a result, no patient underwent unnecessary lateral neck dissection. Since FNA carries little to no morbidity, false-positive ultrasound findings followed by FNA allow for higher sensitivity without increasing danger to the patient. High sensitivity and negative predictive value allow the surgeon to comfortably stage patients at the time of diagnosis without fear of missing metastatic disease.

The specificity of ultrasound in this study (68%) was lower than previously published (80 to 97%) [6, 11, 23, 24]. Positive predictive value (33%) was also lower than previously published (76.5 to 88.8%) [6, 11, 24]. Lower specificity and positive predictive value of ultrasound are acceptable when combined with a liberal use of FNA since negative FNA results reassure the surgeon that metastatic disease is not missed. When results of FNA are added to ultrasound findings, specificity, and positive-predictive value are higher.

Recent publications have compared the utility of US versus computed tomography (CT) versus a combination of both to evaluate the neck in papillary thyroid cancer [9, 23–25]. Both modalities are better at evaluating the lateral neck as compared to the central neck. CT seems to have a potential advantage over ultrasound in evaluation of the central neck [25]. It is common practice at our institution to perform routine central compartment lymph node dissection (CLND) on all patients with a pre- or intraoperative diagnosis of papillary thyroid cancer [26]. When performing routine CLND, the preoperative status of the central lymph nodes as seen on CT scan would not affect surgical planning. Therefore, this potential advantage of CT would provide little utility in our preoperative staging. Furthermore, our sensitivity and negative predictive values are considerably higher than those published for CT and combination of US and CT in these studies (88% versus 62–77%) [23, 24].

## 5. Conclusion

Our results support the current American Thyroid Association recommendations of ultrasound alone as a preoperative staging modality. Furthermore, our data show that surgeon-performed ultrasound of the lateral neck is reliable and equivalent to traditional radiology-performed ultrasound by demonstrating high sensitivity and negative predictive value. By performing ultrasound and FNA in the clinic followed by immediate cytopathologic evaluation, the surgeon has first-hand knowledge of the extent of disease preoperatively. In addition, the patient with papillary thyroid cancer can be examined, diagnosed, and accurately staged all during the same visit.

## Figures and Tables

**Table 1:** Demographics and study results.

Patients included in study	109
Patient characteristics	
Gender	75.5% female
Age	54.1 years (±16.4)
BMI	29.4 (±5.3)
Primary tumor characteristics	
Size	1.85 cm (±1.2)
Multifocality	41%
Lymph node size (evaluated by FNA)	1.37 cm (±0.53)
Average followup	34.1 months (±23.9)
Postoperative RAI scans	92% of patients

**Table 2:** Results of surgeon-performed ultrasound of the lateral neck in papillary thyroid cancer.

Sensitivity	88%
Specificity	68%*
Positive predictive value	33%*
Negative predictive value	97%

*All false-positive results were proven negative by FNA. No patient underwent nontherapeutic lymph node dissection.

## References

[1] Aschebrook-Kilfoy B, Ward MH, Sabra MM, Devesa SS (2011). Thyroid cancer incidence patterns in the United States by histologic type, 1992–2006. *Thyroid*.

[2] Leenhardt L, Grosclaude P (2011). Epidemiology of thyroid carcinoma over the world. *Annales d'Endocrinologie*.

[3] Hughes DT, Haymart MR, Miller BS, Gauger PG, Doherty GM (2011). The most commonly occurring papillary thyroid cancer in the United States is now a microcarcinoma in a patient older than 45 years. *Thyroid*.

[4] Cooper DS, Doherty GM, Haugen BR (2009). Revised American thyroid association management guidelines for patients with thyroid nodules and differentiated thyroid cancer. *Thyroid*.

[5] Marshall CL, Lee JE, Xing Y (2009). Routine pre-operative ultrasonography for papillary thyroid cancer: effects on cervical recurrence. *Surgery*.

[6] Stulak JM, Grant CS, Farley DR (2006). Value of preoperative ultrasonography in the surgical management of initial and reoperative papillary thyroid cancer. *Archives of Surgery*.

[7] González HE, Cruz F, O’Brien A (2007). Impact of preoperative ultrasonographic staging of the neck in papillary thyroid carcinoma. *Archives of Otolaryngology*.

[8] Park JS, Son KR, Dong GN, Kim E, Kim S (2009). Performance of preoperative sonographic staging of papillary thyroid carcinoma based on the sixth edition of the AJCC/UICC TNM classification system. *American Journal of Roentgenology*.

[9] Choi YJ, Yun JS, Kook SH, Jung EC, Park YL (2010). Clinical and imaging assessment of cervical lymph node metastasis in papillary thyroid carcinomas. *World Journal of Surgery*.

[10] Moreno MA, Agarwal G, De Luna R (2011). Preoperative lateral neck ultrasonography as a long-term outcome predictor in papillary thyroid cancer. *Archives of Otolaryngology*.

[11] Hwang HS, Orloff LA (2011). Efficacy of preoperative neck ultrasound in the detection of cervical lymph node metastasis from thyroid cancer. *Laryngoscope*.

[12] Rozycki GS (1998). Surgeon-performed ultrasound: its use in clinical practice. *Annals of Surgery*.

[13] Wilkinson JB, Boyle T, Song J, Kilbride K, Miltenburg D (2008). Surgeon-performed ultrasound reliably predicts skin spacing and may decrease the rate of MammoSite balloon catheter explantation in patients undergoing brachytherapy for breast cancer. *American Journal of Surgery*.

[14] Lindelius A, Törngren S, Pettersson H, Adami J (2009). Role of surgeon-performed ultrasound on further management of patients with acute abdominal pain: a randomised controlled clinical trial. *Emergency Medicine Journal*.

[15] Suthers SE, Albrecht R, Foley D (2004). Surgeon-directed ultrasound for trauma is a predictor of intra-abdominal injury in children. *American Surgeon*.

[16] Soundappan SVS, Holland AJA, Cass DT, Lam A (2005). Diagnostic accuracy of surgeon-performed focused abdominal sonography (FAST) in blunt paediatric trauma. *Injury*.

[17] Solorzano CC, Carneiro DM, Ramirez M, Lee TM, Irvin GL (2004). Surgeon-performed ultrasound in the management of thyroid malignancy. *American Surgeon*.

[18] Prasannan S, Davies G, Bochner M, Kollias J, Malycha P (2007). Minimally invasive parathyroidectomy using surgeon-performed ultrasound and sestamibi. *ANZ Journal of Surgery*.

[19] Méndez W, Rodgers SE, Lew JI, Montano R, Solórzano CC (2008). Role of surgeon-performed ultrasound in predicting malignancy in patients with indeterminate thyroid nodules. *Annals of Surgical Oncology*.

[20] Jabiev AA, Ikeda MH, Reis IM, Solorzano CC, Lew JI (2009). Surgeon-performed ultrasound can predict differentiated thyroid cancer in patients with solitary thyroid nodules. *Annals of Surgical Oncology*.

[21] Milas M, Stephen A, Berber E (2005). Ultrasonography for the endocrine surgeon: a valuable clinical tool that enhances diagnostic and therapeutic outcomes. *Surgery*.

[22] Jabiev AA, Lew JI, Solorzano CC (2009). Surgeon-performed ultrasound: a single institution experience in parathyroid localization. *Surgery*.

[23] Ahn JE, Lee JH, Yi JS (2008). Diagnostic accuracy of CT and ultrasonography for evaluating metastatic cervical lymph nodes in patients with thyroid cancer. *World Journal of Surgery*.

[24] Kim E, Park JS, Son KR, Kim JH, Jeon SJ, Na DG (2008). Preoperative diagnosis of cervical metastatic lymph nodes in papillary thyroid carcinoma: comparison of ultrasound, computed tomography, and combined ultrasound with computed tomography. *Thyroid*.

[25] Choi JS, Kim J, Kwak JY, Kim MJ, Chang HS, Kim EK (2009). Preoperative staging of papillary thyroid carcinoma: comparison of ultrasound imaging and CT. *American Journal of Roentgenology*.

[26] Sadowski BM, Snyder SK, Lairmore TC (2009). Routine bilateral central lymph node clearance for papillary thyroid cancer. *Surgery*.

